# CMOS-Compatible Ultralow-Loss Three-Step Silicon Edge Coupler with Substrate Substitution in the Whole Communication Band

**DOI:** 10.3390/mi14010066

**Published:** 2022-12-27

**Authors:** Zhen Wang, Jin Zhang, Lei Zhang, Xiaoke Ruan, Weijie Tang, Tao Chu

**Affiliations:** 1 Research Institute of Intelligent Networks, Zhejiang Laboratory, Hangzhou 311100, China; 2 College of Information Science and Electronic Engineering, Zhejiang University, Hangzhou 310027, China

**Keywords:** edge coupler, three-step silicon, substrate substitution, taper shape, ultrawide bandwidth

## Abstract

Edge coupler is a key component of silicon-based optoelectronic chips, which dramatically reduces the coupling loss between fibers and transmission waveguides. Here, we propose an ultralow-loss three-step silicon edge coupler based on a 130 nm CMOS process. By replacing the silicon substrate with a material with a lower refractive index than silicon oxide, the silicon leakage loss and polarization-dependent loss can be significantly improved. This structure avoids the existence of a cantilever, which enhances the mechanical strength of the edge coupler. Coupling with standard single-mode fiber, the simulation results demonstrate that the TE/TM mode has an ultralow loss of 0.63/1.08 dB at 1310 nm and 0.57/1.34 dB at 1550 nm, and the 0.5 dB bandwidth covering the entire communication band is about 400 nm. In the entire communication band, the polarization-dependent loss is less than 0.8 dB. Furthermore, we propose a taper shape design method based on mode analysis, which can be adapted for any taper to improve its compactness. Compared with the parabolic shape, the coupling loss of the edge coupler with a length of 460 μm for the TE mode is improved by 0.3 dB on average, this edge coupler provides a feasible solution for fiber-to-chip coupling and is perfectly suitable for wavelength division multiplexing applications in optical communications.

## 1. Introduction

To meet the strong demand for high-speed, large-capacity, and low-power consumption data communication and computing, photonic integrated circuits (PIC) have drawn the great attention from researchers in this field. Benefiting from the high refractive index, low cost, and mature CMOS processing technology, silicon (Si) becomes the preferred choice for the research of PIC. This promotes the development of silicon-based optoelectronics [1,2]. One of the bottlenecks in silicon-based optoelectronics is the low-loss fiber-to-chip coupling [3]. To address this problem, two schemes, grating, and edge coupling have been proposed. Although grating coupling has the advantages of wafer-scale testing, compact size, and large alignment tolerances, it is limited by issues such as relatively large coupling loss, narrow bandwidth, and strong polarization sensitivity. In order to compensate for these shortcomings, edge coupling has attracted more and more attention.

In general, the edge coupling losses are caused by mode mismatch at the fiber-chip interface, taper width variation, silicon leakage, polarization dependence, and so on. Coupling with standard single-mode fibers (SMF), the performance of silicon-based edge coupler is largely restricted by the thickness (typically 220 nm) and minimum width of the waveguide due to the mode mismatch at the fiber-chip interface. In order to address this problem, a silicon nitride (SiN) assistant structure was proposed [4,5]. However, this structure requires additional processing and high alignment accuracy. More importantly, it is also partly affected by the issue of the minimum size of the silicon waveguide for better SiN-Si coupling. In addition, structures assisted by three-dimensional polymer tapers [6] or cascaded silicon oxide (SiO_2_) tapers [7], and double-patterning structures [8] have also been employed. However, these structures are limited by low CMOS compatibility or complex processing. Although the two-step silicon structures [9] and multi-tip structures [10] can partially alleviate this problem, they still face large losses when coupled with standard SMFs. In recent years, most CMOS foundries have developed a mature three-times silicon etching process, including 70-nm, 60-nm, and 90-nm sequential etching. The significant progress in processing technology has brought great opportunities to completely solve the above problems. Another important issue is silicon leakage due to the fact that silicon has a larger refractive index than silicon oxide, which is also related to polarization dependence. A common solution is to introduce cantilever structures [4,7,11]. However, it severely reduces the mechanical strength of the edge coupler, which causes the device to fail under impact. In order to reduce the coupling loss caused by changes in the width or the total length of the taper, various shapes have been proposed, including parabolic [12], segmented linear [13], and constant-loss lines [14]. However, they face either lack of theoretical instruction or difficulty in implementation.

In this paper, we propose a three-step silicon edge coupler based on a 130 nm CMOS process. Benefits from the significant advances in processing technology, this structure can significantly improve the performance of coupling with standard SMFs. To further reduce the losses caused by silicon leakage and polarization dependence, A substrate substitution of a lower-refractive-index material (LRIM) than silicon oxide was introduced, which also improved the mechanical strength of the edge coupler compared with cantilever structures. Based on these improvements, the simulation results show an ultralow loss of 0.63/1.08 dB for TE/TM mode at 1310 nm, 0.57/1.34 dB at 1550 nm, and a 0.5-dB bandwidth of ~400 nm covering the entire communication band. The polarization-dependent loss (PDL) is less than 0.8 dB in the whole communication band. Moreover, we propose a taper shape design method based on mode analysis, which is easy to implement and improves the losses of the TE mode by an average of 0.3 dB compared with the parabolic one with a length of 460 μm.

## 2. Structures and Methods

**Figure 1:**
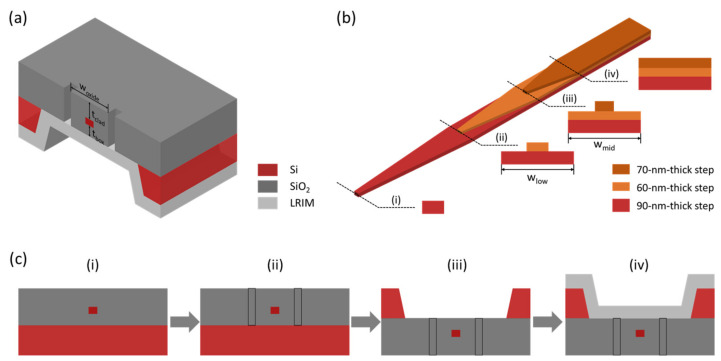
(**a**) Schematic of the proposed three-step silicon edge coupler. (**b**) Details of the three-step silicon taper. (**c**) The processing flow of the proposed edge coupler.

The schematic of the proposed three-step silicon edge coupler is shown in Figure 1a. In this edge coupler, a three-step silicon taper is encapsulated by silicon oxide with a buried oxide (BOX) thickness *t_BOX_* of 3 μm. The silicon oxide waveguide at the first tens of micrometers surrounding the three-step silicon taper is formed by etching two lateral air trenches. The width of the silicon oxide waveguide *w_oxide_* and the cladding thickness *t_clad_* are two important parameters to optimize to further reduce the loss caused by the mode mismatch at the fiber-chip interface. The silicon substrate beneath the silicon oxide waveguide is replaced by an LRIM whose refractive index is lower than silicon oxide. This substrate replacement can avoid silicon leakage and improve the mechanical strength of this structure. The LRIM can be magnesium fluoride (MgF_2_) or silicon oxide with a certain porosity by controlling the deposition conditions such as temperature, variety, ratio, and flow rate of the gas mixture [15,16,17]. In addition, the LRIM could be replaced by other supporting materials such as metals or distributed bragg reflectors, as what has been conducted in the design of grating couplers [18,19,20,21]. The metals or distributed bragg reflectors could provide similar functions as the LRIM. The details of the three-step silicon taper are shown in Figure 1b. The thicknesses of the three steps are 70, 60, and 90 nm successively from top to bottom, and the tip widths of the steps are determined as 130, 150, and 150 nm, respectively, considering the tradeoff between the coupling efficiency of TM mode and processing difficulty. The cross sections of the jump positions are shown next to it. The lower waveguide widths at the jump positions *w_low_* and *w_mid_* are key parameters optimized to minimize the jump loss, especially for the TM mode. Figure 1c shows the processing flow of the proposed edge coupler in Figure 1a. Firstly, starting from a silicon-on-insulator (SOI) wafer with a BOX thickness of 3 μm, the three-step silicon taper is defined by a three-step silicon etching process, followed by cladding deposition. Secondly, the two air trenches with a length of tens of micrometers are etched to form the silicon oxide waveguide. Then, after the SOI wafer is flipped over, the silicon substrate beneath the three-step silicon taper is etched by a silicon deep etching process. Finally, the LRIM is deposited with adequate thickness to achieve sufficient mechanical strength, which could be accompanied by a silicon substrate thinning process. This completes the fabrication of the edge coupler.

To determine the optimal values of *w_oxide_*, *w_low_*, and *w_mid_*, we calculate the overlap of mode profiles before and after the jump using the finite difference eigenmode (FDE) solver in Lumerical software. The mode profile overlap η is defined as follows:

(1) $$\eta =\frac{{\left|\iint^{\;}{\vec{E}}_{1}\left(x,y\right)\cdot {\vec{E}}_{2}^{*}\left(x,y\right)dA\right|}^{2}}{\iint^{\;}{\left|{\vec{E}}_{1}\left(x,y\right)\right|}^{2}dA\iint^{\;}{\left|{\vec{E}}_{2}\left(x,y\right)\right|}^{2}dA}$$

where ${\vec{E}}_{1}\left(x,y\right)$ and ${\vec{E}}_{2}\left(x,y\right)$ are the mode profiles of the electric field before and after the jump, respectively. In the simulation, all parameters of the edge coupler were optimized at the wavelength of 1310 nm. At this wavelength, the refractive indexes of silicon, silicon oxide, and LRIM are chosen to be 3.50, 1.46, and 1.42, respectively. The cladding thickness was determined to be 6 μm, considering the losses caused by mode mismatch at the fiber-chip interface and transmission. The diameter of the mode profile of a standard SMF is ~9 μm at a wavelength of 1310 nm. The total thickness (BOX plus cladding) of the silicon oxide waveguide is sufficient to possess a high mode profile overlap with standard SMF coupling. And according to our simulation experiences, when the cladding thickness is much larger, the optical power above the three-step silicon taper is difficult to couple into the silicon waveguide. Therefore, a cladding thickness of 6 μm is a trade-off for both minimizing the mode mismatch loss at the fiber-chip interface and the transmission loss.

**Figure 2:**
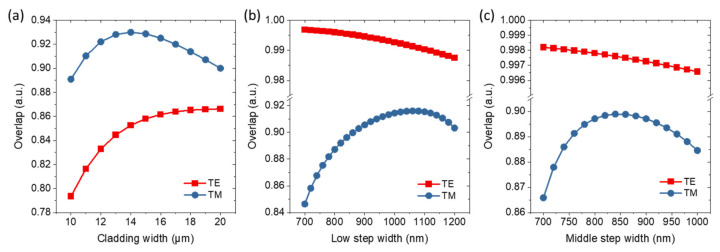
(**a**) Overlap of mode distributions between a standard SMF and the tip of low-step taper at different widths of silicon oxide waveguide *w_oxide_*. Overlap of mode distributions at position (**b**,**c**), as shown in Figure 1b, with different *w_low_* and *w_mid_*.

The mode profile overlaps between a standard SMF and the tip of low-step taper for different widths of silicon oxide waveguide *w_oxide_* are shown in Figure 2a. Over the calculated range of *w_oxide_*, the TM mode profile overlap exhibits a maximum value of ~93% at a width of 14 μm. The TE mode profile overlap is ~85% at a width of 14 μm and gradually saturates after this width. Figure 2b shows the mode profile overlaps at position ii as shown in Figure 1b with different *w_low_*. Within the calculated range of *w_low_*, the TM mode profile overlaps exhibit a maximum value of ~92% at a width of 1060 nm, while the TE mode profile overlaps slightly decrease with a value of ~99%. Likewise, the mode profile overlaps at position iii, as shown in Figure 1b, with different *w_mid_* are shown in Figure 2c. The TM mode profile overlap exhibits a maximum value of ~90% at a width of 840 nm, while the TE mode profile overlap maintains values above 99% over the calculated range of *w_mid_*. Finally, considering improving the overall TM mode coupling efficiency and reducing the polarization-dependent loss, the optimized values of *w_oxide_*, *w_low_*, and *w_mid_* are determined to be 14 μm, 1060 nm, and 840 nm, respectively. The simulated mode profiles and effective refractive indexes, effective areas, and TE polarization fractions of TE and TM modes at positions i, ii, and iii with the final determined parameters are shown in Figure S1 and Table S1 of the supplementary material, respectively. This information helps to explain the different overlap values between TE and TM modes.

**Figure 3:**
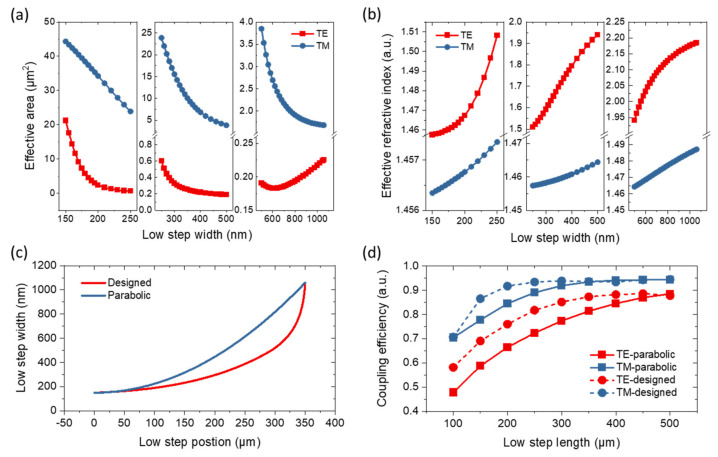
(**a**) Effective refractive indexes and (**b**) areas of TE and TM modes at different widths of low-step silicon taper from 150 to 1060 nm. (**c**) The determined shape of the low-step silicon taper, accompanied by a parabolic one. (**d**) Transmission efficiencies of the low-step silicon taper with different lengths for designed and parabolic shapes.

Although the key parameters such as *t_clad_*, *w_oxide_*, *w_low_*, and *w_mid_* have been determined above, it is important to design the shape of the three-step silicon taper. To achieve adiabatic transmission with the shortest taper length, we propose a design method based on mode analysis. Figure 3a,b show the effective refractive indexes and areas of the TE and TM modes, respectively, for different widths of low-step silicon taper from 150 to 1060 nm. These parameters are also calculated by using FDE solver in Lumerical software. The taper widths are divided into three ranges, namely the ranges of 150–250 nm, 250–500 nm, and 500–1060 nm. In the range of 150–250 nm, the effective refractive indexes change slowly for both TE and TM modes. However, the effective area of the TE mode changes drastically, while that of the TM mode changes relatively slowly. Therefore, we choose the effective area of the TE mode as the optimization object. In the range of 250–500 nm, the effective area of the TM mode and the effective refractive index of the TE mode exhibit relatively rapid changes. Considering improving the transmission efficiency of the TM mode, the effective area of the TM mode is selected as the optimization object. In the range of 500–1060 nm, all four variates change a little with the width. Preferably, the effective area of the TM mode with a larger change is selected as an optimization object. During the optimization process, to minimize the transmission loss and length, the effective areas should change at an equal rate, considering the mode matching at an arbitrary position. Then, we fit the relationship between the effective areas and widths using interpolation. Based on the fitted relationship, the width array corresponding to the effective area array with an equal decreasing rate can be obtained. Finally, the taper shapes of the three width ranges can be determined by associating the obtained width arrays with equally spaced length arrays. 

Based on this design method, the determined shape of the low-step silicon taper is shown in Figure 3c, with a parabolic taper for comparison. The designed taper shape exhibits a slower rate of change at the beginning and a faster rate of change at the end than the parabolic one. To compare the performances of these two taper shapes, we simulate the transmission efficiencies of the low-step silicon taper at different lengths by using FDTD (finite difference time domain) solver in Lumerical software. The results are shown in Figure 3d. For the designed taper shape, the adiabatic-transmission length of the TE mode is ~350 μm with a coupling efficiency of ~87%, while that of the TM mode is ~200 μm with a coupling efficiency of ~92%. However, the adiabatic-transmission lengths of the parabolic one are ~500 and ~350 μm, which are ~43% and ~75% longer than those of the designed taper shape for the TE and TM modes, respectively. Before the adiabatic-transmission lengths of the designed one, the coupling efficiencies were much larger than those of the parabolic one. Therefore, the taper shape design method is well suited for the implementation of an ultra-compact edge coupler. Finally, considering the trade-off between compact size and large coupling efficiency, we choose a length of 350 μm for the low-step silicon taper with the designed shape. The shapes and lengths of the middle and top-step silicon tapers are determined by the same approach. 

## 3. Results and Discussions

**Figure 4:**
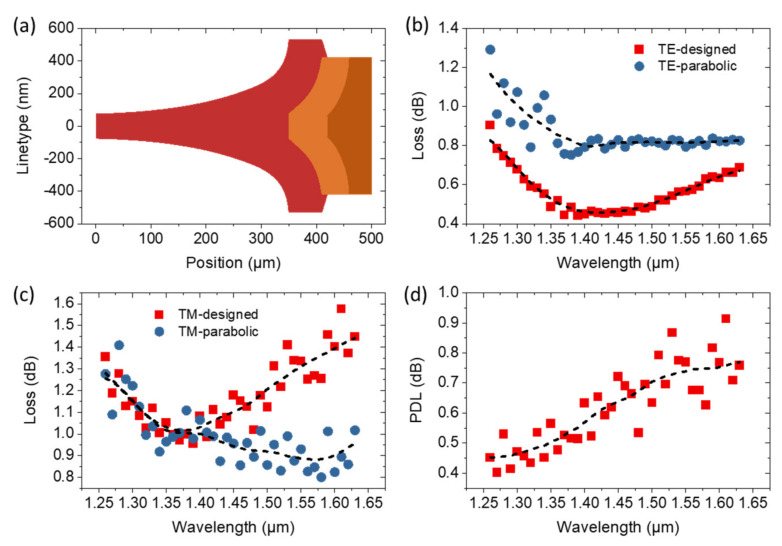
(**a**) Total shape of the three-step silicon taper with a length of 460 μm. Coupling loss spectra of (**b**) TE and (**c**) TM modes accompanied by those using parabolic one across the whole communication band. (**d**) PDL spectra of the edge coupler with the designed line type. Dashed lines in (**b**–**d**) are the smoothed curves of the simulation data.

The total shape of the three-step silicon taper with a length of 460 μm is shown in Figure 4a. Then, the transmission spectra of the edge coupler consisting of the three-step silicon taper shown in Figure 1a were simulated using FDTD solver in Lumerical software. The coupling loss spectra of the TE and TM modes over the entire communication band (1260–1625 nm) accompanied by those using parabolic taper shape are shown in Figure 4b,c, respectively. For the designed taper shape, the coupling loss for TE/TM mode is 0.63/1.08 dB at 1310 nm with a PDL of 0.45 dB. Although the coupling loss of this edge coupler is optimized at 1310 nm, the results at 1550 nm are also competitive with a coupling loss of 0.57/1.34 dB for TE/TM mode, and the results can be further improved by optimization at this wavelength. In addition, the deviation of the coupling loss is less than 0.5 dB across the whole communication band. In other words, the 0.5-dB bandwidth for both TE and TM modes is around 400 nm, which is the best result in the literature and perfectly suitable for wavelength division multiplexing applications in optical communication. Moreover, the coupling loss of this edge coupler with the designed taper shape is improved by 0.3 dB on average for the TE mode compared with that of the parabolic one, while the improvement is neglectable for the TM mode in the range of 1260–1400 nm. Although the TM mode losses of the designed one are larger than those of the parabolic one at longer wavelengths, they can be significantly improved by optimizing in this wavelength range. The PDL spectra of this edge coupler with the designed taper shape are shown in Figure 4d. In the whole communication band, the PDL is less than 0.8 dB.

**Figure 5:**
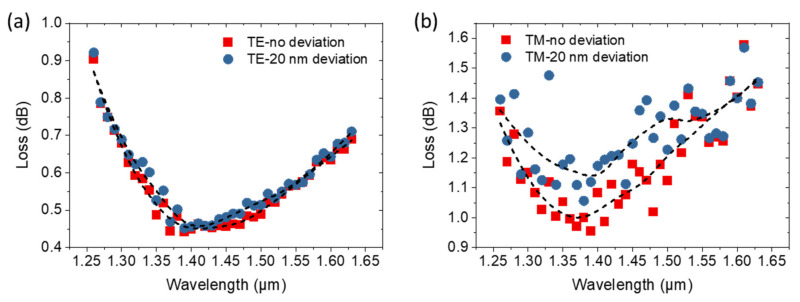
The simulated coupling losses of (**a**) TE and (**b**) TM modes with 20-nm alignment deviation compared to those without deviation.

As mentioned in the processing flow of the proposed edge coupler, the processes of deep silicon etching, silicon substrate thinning, and supporting material deposition are needed to perform. Indeed, the high aspect ratio trenches fabricated by deep silicon etching could affect the supporting material deposition, and the silicon substrate thinning process could affect the final fragility and bow of the chip. However, there are several solutions to eliminate or alleviate these problems. First, the proposed edge coupler can be fabricated without the deposition of supporting material, which can also possess similar coupling performances and stronger mechanical strength than the cantilever structure, as shown in Figure S2 of supplementary material. The deposition of supporting material will further improve the mechanical strength. Second, to deposit the supporting material, the width of the trenches in the backside could be increased properly without the silicon substrate thing or with less. To further verify the experimental feasibility, we simulated the influence of alignment deviation of the three-step silicon taper on the coupling loss. The simulated coupling losses of TE and TM modes with 20-nm deviation compared to those without deviation are shown in Figure 5. The simulated coupling losses of TE mode with 20-nm deviation show negligible change compared to that without deviation, while those of TM mode increase by a maximum value of 0.15 dB. With the 20-nm deviation, the 0.5-dB bandwidth is still ~400 nm, covering the entire communication band. These results indicate the great experimental feasibility of the proposed edge coupler.

To compare the comprehensive performance of this edge coupler with other couplers in the literature, we summarize the results of coupling with standard SMFs in the literature and this work, as shown in Table 1. The minimum coupling loss at 1310 nm in the literature is 2/1.8 dB for TE/TM mode in experiments [22], while 0.95/1.3 dB at 1550 nm [11]. According to the high alignment tolerance of the three-step silicon taper, as shown in Figure 5, an extra 0.5 dB loss due to imperfection in fabrication is taken into account to make a fair comparison with the experimental results in the literature. Thus, the coupling loss of this work in the experiment is estimated to be 1.13/1.58 dB at 1310 nm and 1.07/1.84 dB at 1550 nm, which are comparable to the best results. More importantly, our results show great superiority in terms of bandwidth, minimum width, compactness, and mechanical strength. Although some simulations at 1550 nm without the use of cantilevers show decent coupling losses [23,24], they are difficult to fabricate and offer no advantages in terms of bandwidth and compactness compared to this work. In addition, the coupling loss of the proposed edge coupler in this work at 1550 nm can be further improved by designing at this wavelength. All in all, although the proposed edge coupler in this work has not been experimentally demonstrated, it exhibits competitive coupling losses compared to the results in the literature and can be fabricated with a 130 nm CMOS process. Moreover, our results possess ultrawide bandwidth, excellent compactness, and strong mechanical strength, showing great potential to thoroughly solve the problems in fiber-to-chip coupling.

## 4. Conclusions

In summary, we propose, design, and simulate a three-step silicon edge coupler with substrate substitution, which can be fabricated in a 130 nm CMOS process. By replacing the silicon substrate with LRIM, the silicon leakage loss is dramatically prevented, and the mechanical strength is significantly improved compared with the cantilever structure. Moreover, we propose a taper shape design method based on mode analysis, which can significantly reduce the overall length of the edge couplers compared with parabolic ones. Coupled with a standard SMF, the proposed edge coupler with a length of 460 μm exhibits ultralow losses of 0.63/1.08 dB for TE/TM mode at 1310 nm, 0.57/1.34 dB at 1550 nm, a 0.5-dB bandwidth of ~400 nm covering the entire communication band. The TE mode is improved by an average of 0.3 dB compared to the parabolic shape., The PDL is less than 0.8 dB in the whole communication band. Compared with the edge couplers in the literature, our results show competitive coupling loss and great superiority in terms of bandwidth, minimum width, compactness, and mechanical strength. The proposed edge coupler is perfectly suitable for wavelength division multiplexing applications in optical communication and has great potential to completely solve the problems of fiber-to-chip coupling.

## Figures and Tables

**Table 1:** Performances of the edge couplers coupling with standard SMFs in the literature and this work.

Year	Structure	Wavelength (nm)	S/E * TE Loss (dB)	S/E * TM Loss (dB)	S/E * PDL (dB)	Loss-Bandwidth (dB-nm)	Minimum Width (nm)	Length (μm)	Cantilever
2016 [7]	Cascaded SiO_2_ to two-step Si	1550	--/1.6	--/2.3	--/0.7	0.5–100	100 (Si)	240	yes
2016 [22]	SiN array to Si	1310 1550	--/2 --/1.75	--/1.8 --/1.6	--/0.2 --/0.15	0–40 1–100	80 (Si)	--	no
2017 [4]	SiN-Si coupling	1310 1550	--/2.8 --/2.9	--/3 --/3.4	--/0.2 --/0.5	0.5–100 1–100	150 (SiN)	300 (SiN)	yes
2018 [11]	SiO_2_ cantilever to two-step Si	1550	0.5/0.95	1/1.3	0.5/0.35	0.2–100	105 (Si)	--	yes
2019 [23]	SiN/SiO_2_ stack to Si	1550	0.4/--	0.43/--	0.03/--	0.6–100	150 (Si)	1870	no
2019 [25]	Si with air trench	1310	1.1/2	--	--	1–60	70 (Si)	130	no
2021 [26]	Trident Si with SWG **	1550	0.9/2.22	1.3/2.53	0.4/0.31	1–120	130 (Si)	90	no
2022 [5]	SiN-Si coupling	1550	--/1.55	--/1.65	--/0.1	1–80	80 (SiN) 80 (Si)	550	yes
2022 [24]	SiN array to Si	1550	0.49/--	0.92/--	0.43/--	1–160	200 (SiN) 150 (Si)	1200	no
This work	Three-step Si	1310 1550	0.62/-- 0.56/--	1.11/-- 1.33/--	0.49/-- 0.77/--	0.5–400	130	460	no

* S/E means simulated/experimental. ** SWG means subwavelength grating.

## Data Availability

The data presented in this study are available on request from the corresponding author. The data are not publicly available due to privacy.

## Supplementary Materials

The following supporting information can be downloaded at: https://www.mdpi.com/article/10.3390/mi14010066/s1, Figure S1: The simulated mode profiles of TE and TM modes at positions i, ii, and iii with the final determined structural parameters; Table S1: The simulated effective refractive indexes (n), effective areas (A) and TE polarization fractions (η) of TE and TM modes at positions i, ii, and iii with the final determined structural parameters; Figure S2: The simulated total deformations of the (a) proposed structure without the supporting material and (b) cantilever structure under a force of 1 N on the facet, while (c) and (d) under a pressure of 10 MPa on the top surface.
