# Supplementary material for: CMOS-Compatible Ultralow-Loss Three-Step Silicon Edge Coupler with Substrate Substitution in the Whole Communication Band
Source: Micromachines (Basel). 2022 Dec 27;14(1):66. doi: 10.3390/mi14010066 (PMC9861178; doi:10.3390/mi14010066)

Supplementary Material for

CMOS-compatible ultralow-loss three-step silicon edge coupler with substrate substitution in the whole communication band

Zhen Wang[1], Jin Zhang[1], Lei Zhang[1], Xiaoke Ruan[1], Weijie Tang[1], and Tao Chu[1,2,*]

1 Research Institute of Intelligent Networks, Zhejiang Laboratory, Hangzhou 311100, China

2 College of Information Science and Electronic Engineering, Zhejiang University, Hangzhou 310027, China

* Correspondence: chutao@zju.edu.cn

Figure S1 The simulated mode profiles of TE and TM modes at positions i, ii, and iii with the final determined structural parameters.

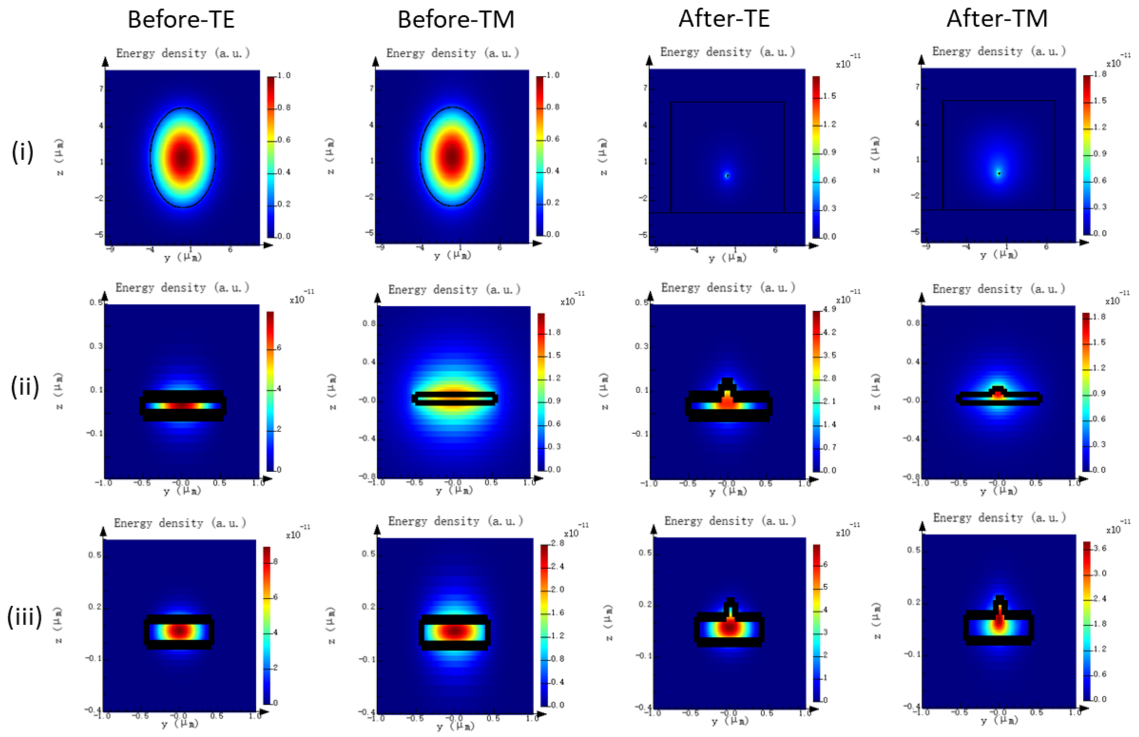

Table S1 The simulated effective refractive indexes (n), effective areas (A) and TE polarization fractions (η) of TE and TM modes at positions i, ii, and iii with the final determined structural parameters.

	Before-TE			Before-TM			After-TE			After-TM		
	n (a.u.)	A (μm^2)	η (%)	n (a.u.)	A (μm^2)	η (%)	n (a.u.)	A (μm^2)	η (%)	n (a.u.)	A (μm^2)	η (%)
i	1.437532	60.2438	100	1.437591	63.8369	0	1.457583	21.191	100	1.456345	44.3238	0
ii	2.185184	0.225246	100	1.487259	1.68312	0	2.266955	0.185257	99	1.528383	0.766938	2
iii	2.597318	0.163905	100	1.678703	0.439667	2	2.643663	0.158301	99	1.868711	0.276035	5

Figure S2 The simulated total deformations of the (**a**) proposed structure without the supporting material and (**b**) cantilever structure under a force of 1 N on the facet, while (**c**) and (**d**) under a pressure of 10 MPa on the top surface.

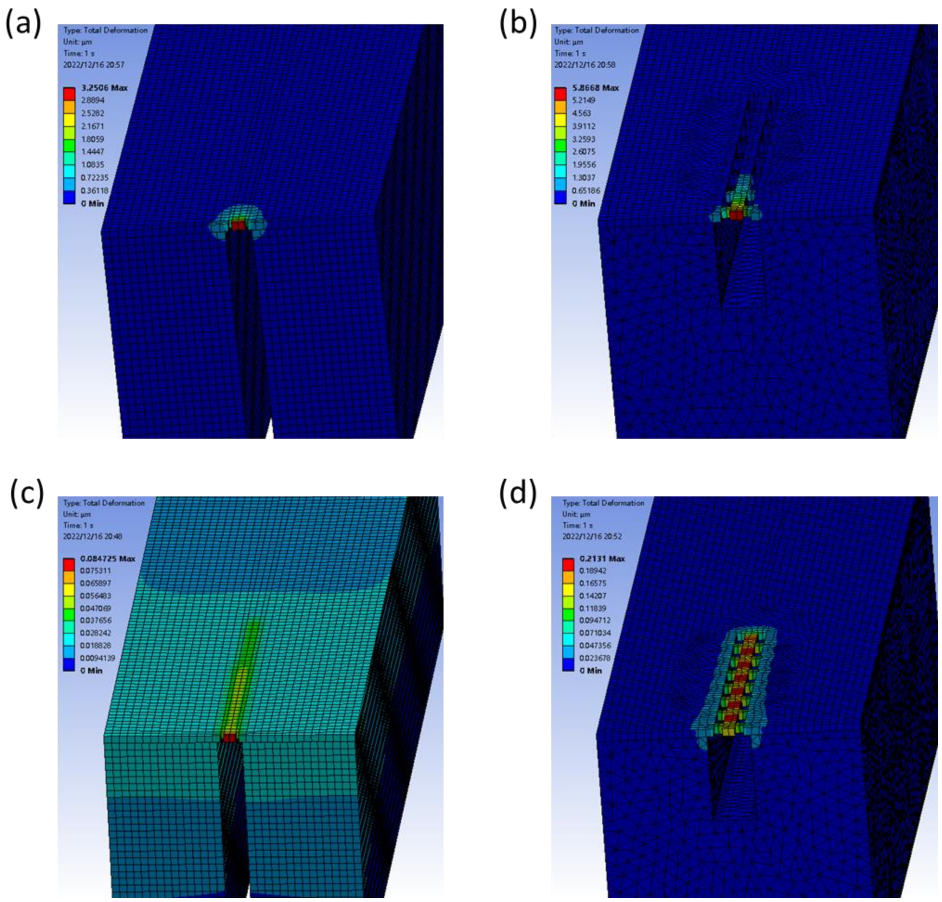

Supplement: Supplementary file 1 [file micromachines-14-00066-s001.zip › micromachines-2083437-supplementary.pdf]
